# The release patterns and potential prebiotic characteristics of soluble and insoluble dietary fiber-bound polyphenols from pinot noir grape pomace *in vitro* digestion and fermentation^☆^

**DOI:** 10.1016/j.fochx.2025.102694

**Published:** 2025-06-21

**Authors:** Yuanyuan Li, Xiaoxue Chen, Gongda Wang, Linting Xu, Yichen Liu, Chunlong Yuan, Junjun Li

**Affiliations:** a College of Enology, Northwest A&F University, Yangling, 712100, Shaanxi, China; b Shaanxi Provincial Key Laboratory of Viti-Viniculture, Yangling, 712100, Shaanxi, China; c Chateau Junding Co. Ltd., Penglai, 265607, China

**Keywords:** Grape pomace, Soluble dietary fiber, Insoluble dietary fiber, Bound polyphenols, Bioactivity, *In vitro* digestion, Gut microbiota

## Abstract

The bioactive substances abundant in grape pomace, particularly dietary fiber and polyphenols, exhibit prebiotic effects in the gut. The soluble dietary fiber and insoluble dietary fiber from the Pinot noir grape pomace were subjected to *in vitro* simulated digestion and colonic fermentation. The study found that bound polyphenols predominantly released during colon fermentation and exerted powerful antioxidant activities. The capabilities of SDF were superior to those of IDF in several aspects: it more effectively released total phenols and flavonoids, scavenged ABTS and hydroxyl radicals, inhibited α-glucosidase and lipase, regulated intestinal pH, and generated short-chain fatty acids. IDF enriched the diversity of the gut microbiota. Together, SDF and IDF reduced the F/B value, promoted beneficial bacteria, inhibited harmful bacteria, activated metabolic pathways for vitamin B6, ascorbate, and aldarate, and enhanced lipopolysaccharide and folate biosynthesis. These findings provide a basis for the development of dietary fiber in grape pomace as a functional food.

## Highlights

• Dietary fiber bound polyphenols (DFP) are released and metabolized in colon fermentation.
• Soluble dietary fiber demonstrates superior potential bioactivity *vs.* insoluble.
• DFP boosts probiotics (*e.g.*, *Bifidobacterium longum*) and inhibits pathogens.
• Dietary fiber regulates intestinal pH and boosts SCFAs like propionic acid.
• DFP enhances gut flora richness, and insoluble dietary fiber boosts α-diversity.

## Graphical abstract

**Figure:**
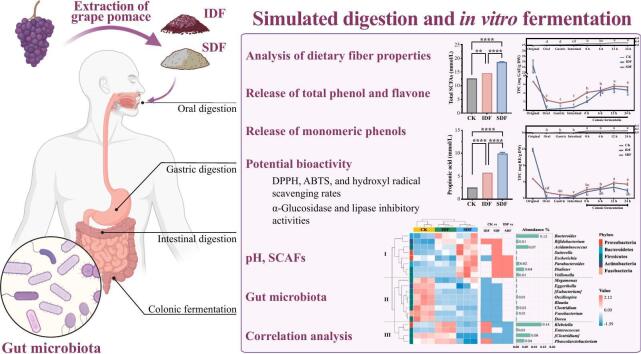

## 1 Introduction

Grape pomace, a by-product of wine production, is a potential source of prebiotics in functional foods due to its high dietary fiber (DF) content (Beutinger et al., 2020). DF can be classified into soluble DF (SDF) and insoluble DF (IDF) based on its water solubility. SDF mainly consists of soluble hemicelluloses, pectin, and oligosaccharides, whereas IDF comprises cellulose, insoluble hemicelluloses, and lignin (Liu et al., 2024). Both SDF and IDF play vital roles in promoting human health by reducing blood glucose, lipid, and cholesterol levels, as well as lowering the risk of cardiovascular and intestinal diseases (Dhingra et al., 2012). Additionally, grape pomace is a rich source of bioactive polyphenols, including anthocyanins, procyanidins, flavonols, resveratrol, and phenolic acids, which exert antioxidant, anti-inflammatory, blood pressure–lowering, and cardioprotective effects (Carullo et al., 2020).

A substantial proportion of phenolic compounds in grape pomace are covalently bound to DF through ester, ether (87 %), or glycosidic bonds (13 %) (Maurer et al., 2019). After ingestion, DF transports bound polyphenols, undergoing modifications in the gastrointestinal tract and subsequent fermentation in the colon. This results in the production of bioactive metabolites such as short-chain fatty acids (SCFAs) and phenolic acids under the influence of gut microbiota (Del Rio et al., 2013). These metabolites continuously scavenge free radicals, reduce intestinal pH, and suppress the growth of pathogenic bacteria, thus maintaining a healthy colonic environment (Louis et al., 2014). Moreover, these metabolites are absorbed through the colon, where they trigger anti-inflammatory responses and play a crucial role in preventing diseases such as colorectal cancer (Pérez-Ortiz et al., 2019). Digested and fermented DF exhibits a synergistic effect with bound polyphenols, displaying a higher antioxidant capacity compared with polyphenols alone. Bound antioxidants in insoluble food matrices (especially DF) can be regenerated through colonic fermentation (Çelik et al., 2013). Bound polyphenols become antioxidant radicals by releasing electrons or hydrogen atoms to quench free radicals. In contrast, the gastrointestinal microbiota releases soluble phenolic compounds as hydrogen donors to restore their antioxidant activity. These findings indicate that DF-bound polyphenols (DFPs) possess strong antioxidant activity and prebiotic characteristics. Therefore, exploring the role of metabolites released and transformed during the digestion and colonic fermentation of DF is particularly important.

Research on DF in grape pomace primarily focuses on improving and innovating extraction methods (Cui et al., 2023; Martínez-Meza et al., 2021). Studies evaluating the content, transformation, bioactivity of bound polyphenols released after *in vitro* digestion and fermentation of DF, and the impact of DF on the gut microbiota also mainly focus on other materials such as coffee, mangosteen peel, and wheat bran (Sun et al., 2024; Wu et al., 2022; Zhang et al., 2023), with less research using grape pomace as raw material. Furthermore, a current lack of synchronous comparison exists between the prebiotic activity and functional differences of IDF and SDF in grape pomace during digestion and fermentation. Research using the Pinot noir grape variety as raw material is even scarce. Therefore, this study was conducted to investigate the release patterns of bound polyphenols from IDF and SDF during simulated *in vitro* digestion and fermentation, as well as their potential biological activities, and the modulatory impact of IDF and SDF on gut microbiota composition and metabolic pathways. The study findings provide valuable insights into the use of grape pomace polyphenol–fiber complexes for enhancing gut health through their synergistic effects and promoting the growth of beneficial microbiota.

## 2 Materials and methods

### 2.1 Materials and reagents

Pinot noir grape pomace was donated by Bainianzhuang Wine Industry Co., Ltd. (Xinjiang Uygur Autonomous Region, China). The grape skins were selected from the grape pomace. They were first dried by air-drying and then in an oven at 40 °C until no significant difference in weight was observed between the two weightings. Next, they were ground into a powder form and then sieved through a mesh with an 80-μm size to obtain a fine powder.

Glucoamylase, α-amylase (from porcine pancreas, 14 U/mg), papain, lipase (from porcine pancreas), pepsin (from porcine gastric mucosa, 15,000 U/mg), pancreatin (from porcine pancreas, 250 U/mL), and bile salts (from porcine bile) were purchased from Shanghai Aladdin Biochemical Technology Co., Ltd. (Shanghai, China).

For determining the phenolic content, antioxidant activity, and enzyme-inhibition rate, the following reagents and standards were purchased from Shanghai Source Leaf Biotechnology Co., Ltd. (Shanghai, China): 2,2-diphenyl-1-picrylhydrazyl (DPPH), 2,2′-azino-bis-(3-ethylbenzothiazoline-6-sulfonic acid) (ABTS) for antioxidant assays; salicylic acid, Folin–Ciocalteu reagent, gallic acid, and rutin for phenolic content analysis; 4-nitrophenol-α-D-glucopyranoside (*p*-NPG) and *p*-nitrophenyl laurate (*p*-NPL) for enzyme inhibition assays; individual phenolic standards for HPLC quantification, and chromatographic-grade methanol and ethanol for sample preparation and HPLC analysis. Deionized water was obtained by using the Milli-Q System (Millipore, MA, USA). All other chemicals used in this study were of analytical grade.

### 2.2 Preparation of SDF and IDF from pinot noir pomace

SDF and IDF were prepared by a previously reported method, albeit with some modifications (Wang et al., 2022). Briefly, 1 g of grape skin powder was mixed with 40 mL of distilled water, to which 5 μL of 0.25 % (*w*/*v*) α-amylase (pH 6.0, 70 °C, 1 h) was added. Then, 25 μL of 5 % papain (*w*/*v*) (pH 6.0, 60 °C, 1 h) was added, followed by the addition of 20 μL of 20 % glycosidase (*w*/*v*) (pH 4.5, 60 °C, 30 min) with constant stirring. The mixture was centrifuged at 9500*g* for 15 min and washed twice with ethanol. The residue was collected and freeze-dried to obtain IDF. Meanwhile, the supernatant obtained from the first centrifugation was evaporated at 50 °C before adding four volumes of ethanol, allowed to stand overnight at 4 °C, centrifuged at 9500*g* for 15 min), and washed twice with ethanol. The precipitate was separated and freeze-dried to obtain the SDF.

### 2.3 Simulated *in vitro* digestion and colonic fermentation

*In vitro* fermentation was conducted using a previously reported method (Li et al., 2024), albeit with some modifications. Fig. S1 shows the schematic diagram of the main steps and sample collection of simulated *in vitro* digestion and colon fermentation. Briefly, the SDF or IDF samples were first mixed with simulated salivary fluid (SSF) at 37 °C. Then, α-amylase was added as the main digestive component for simulated oral digestion, followed by mixing with an equal volume of simulated gastric fluid (SGF) preheated to 37 °C, initiating the gastric digestion stage with pepsin as the digestive component. Next, the samples were mixed with an equal volume of simulated intestinal fluid (SIF) at 37 °C to initiate the intestinal digestion stage, with pancreatin and bile as the main digestive components. For the specific components and the additional amounts of the electrolyte stock solutions for SSF, SGF, and SIF, please refer to the INFOGEST 2.0 protocol published by Brodkorb et al. (2019). The digests from the different digestion stages of the reaction were centrifuged at 9500*g* for 10 min, and the supernatants were taken to obtain samples of the digests from each stage.

The final stage was the *in vitro* fermentation of human fecal microbiota. We collected fresh fecal samples from four healthy volunteers who had no intestinal diseases and had not used antibiotics in the last 3 months. Then, equal amounts of fecal samples were mixed and diluted with saline, and the supernatant was collected after centrifugation. The supernatant was then added to the basal culture medium containing intestinal digestion residues and cultured anaerobically at 37 °C. The fermentation broth was collected after 0, 6, 12, and 24 h and centrifuged at 9500*g* for 10 min. The supernatant was separated to measure the pH value. In the aforementioned stages, the control (CK) group was replaced with an equal volume of water instead of the sample, while the remaining steps remained unchanged. All samples were stored in a refrigerator at −80 °C for subsequent analyses.

### 2.4 Determination of the phenolic contents

The total phenol content (TPC) in all samples was determined using the Folin–Ciocalteu reagent. The results were expressed as milligrams of gallic acid equivalent (GAE) per 1 g of dry weight (mg GAE/g DW). The total flavone content was determined by the NaNO_2_–AlCl_3_ method. The results were expressed as milligrams of rutin equivalent per 1 g of dry weight (mg/g DW).

A total of 14 monomer phenols were quantified using the ultra-high-performance liquid chromatography system (Shimadzu Corporation, Japan). Separation was performed on a C18 column (100 × 2.1 mm^2^, 2 μm) using a mobile phase composed of water with 0.1 % formic acid (A) and 100 % acetonitrile (B). The flow rate was set to 0.3 mL/min, with an injection volume of 1 μL, and the column temperature was set to 30 °C. The gradient elution program was as follows: 0–5 min, 8 %–10 % B; 5–8 min, 10 %–13 % B; 8–10 min, 13 %–20 % B; 10–15 min, 20 %–15 % B; 15–17 min, 15 %–34 % B; 17–19 min, 34 %–45 % B; 19–24 min, 45 %–8 % B. The chromatogram was observed and treated at 280 nm, and the monomer phenols were identified by comparing the retention time.

### 2.5 Determination of the antioxidant activity

DPPH, ABTS (Chen et al., 2024), and hydroxyl radical–scavenging activities (Zhang et al., 2024) were determined to assess the antioxidant capacity of all samples at the digestion and fermentation stages.

DPPH radical–scavenging activity: First, 40 μL of the sample was mixed with 160 μL of 0.1 mmol/L DPPH. The absorbance at 517 nm was recorded using a microplate reader (Thermo Scientific Varioskan LUX, DE, USA) after the solution was placed in the dark for 30 min, which was labeled as *A*_1_. Then, 100 % methanol was used to replace the sample, and the absorbance measured was labeled as *A*_0_. Further, 40 μL of the sample was mixed with 160 μL of 100 % methanol, and the absorbance measured was labeled as *A*_2_.

ABTS radical–scavenging activity: First, the ABTS solution was prepared by adding an appropriate amount of K_2_S_2_O_8_ to the already prepared 7 mmol/L ABTS solution to reach a concentration of 2.45 mmol/L. The solution was stored in the dark for 12 h before use. Then, 15 μL of the sample was mixed with 1985 μL of the ABTS solution and allowed to stand in the dark for 6 min. The absorbance *A*_1_ was recorded at 734 nm. The sample was then replaced with 100 % methanol to measure the absorbance *A*_0_. Next, 15 μL of the sample was mixed with 1985 μL of 100 % methanol to measure the absorbance *A*_2_.

Hydroxyl radical–scavenging activity: Equal volumes (100 μL) of FeSO_4_ (9 mmol/L), salicylic acid–ethanol solution (9 mmol/L), H_2_O_2_ (8.8 mmol/L), and the sample were mixed and incubated at 37 °C for 30 min. The absorbance *A*_1_ was recorded at 510 nm. Distilled water was used as a reference to measure the absorbance *A*_0_. Distilled water was used instead of H_2_O_2_ to measure the absorbance *A*_2_.

The DPPH, ABTS, and hydroxyl radical–scavenging rates were calculated using Eq. (1):

(1) $$\text{Radical scavenging rate}\;\left(\%\right)=\left(1-\frac{A_{1}-A_{2}}{A_{0}}\right)\times 100\%$$

### 2.6 Determination of the enzyme-inhibitory activity

The enzyme-inhibitory activity was evaluated using α-glucosidase (Guo et al., 2023) and lipase (Manzoor et al., 2021) as described previously, with some modifications.

α-Glucosidase inhibition assay: First, 30 μL of the sample was mixed with 20 μL of the α-glucosidase solution (0.25 U/mL, prepared in advance in 0.1 M sodium phosphate buffer pH 6.9) and then incubated in a water bath at 37 °C for 10 min. Then, 50 μL of 2 mmol/L *p*-NPG was added to initiate the reaction. After 20 min, 150 μL of Na_2_CO_3_ was added to terminate the reaction. A microplate reader was used to record the absorbance *A*_1_ at 405 nm. The absorbance *A*_2_ was recorded when using reagents for phosphate-buffered saline (PBS) instead of α-glucosidase. PBS was used as a substitute for the sample, and the absorbance was recorded as *A*_0_. PBS was used to replace both α-glucosidase and the sample, and the absorbance was recorded as *A*_3_.

Lipase-inhibition assay: A lipase stock solution with a concentration of 5 mg/mL was prepared with 0.1 mol/L Tris-HCl buffer. *p*-NPL was dissolved in a 5 mmol/L sodium acetate solution (containing 1 % Triton X-100) in a boiling water bath. Then, 15 μL of the sample was mixed with 85 μL of the lipase stock solution and incubated in a water bath at 37 °C for 15 min. Further, 100 μL of *p*-NPL was added to initiate the reaction and incubated for an additional 45 min. A microplate reader was used to record the absorbance *A*_1_ at 405 nm. The absorbance of the system without lipase was recorded as *A*_2_, the absorbance of the system without the sample was recorded as *A*_0_, and the absorbance of the system without both the sample and lipase was recorded as *A*_3_.

The inhibition rates for α-glucosidase and lipase activities were calculated using Eq. (2):

(2) $$\begin{array}{c} \text{Inhibition rate}\;\left(\%\right)=\left(1–\frac{A_{1}–A_{2}}{A_{0}–A_{3}}\right)\times 100\% \end{array}$$

### 2.7 Content determination of SCFAs

The contents of SCFAs were determined by a previously described method (Li et al., 2024). For this, 2-ethyl butyric acid was used as an internal standard and added to 1 mL of the fermentation broth. The sample was separated on a DB-FFAP column (30 m × 0.25 mm × 0.25 μm; Agilent, USA) using a gas chromatograph (GC-2014; Shimadzu Corporation).

### 2.8 Gut microbiota analysis

The final fermentation broth was handed over to Shanghai Personal Biotechnology Co., Ltd. (Shanghai, China) for PE250 paired-end sequencing on the Illumina NovaSeq platform. The QIIME2 software was used to analyze the biological information of the microbiome and compare it with the Greengenes database to obtain the taxonomic information.

### 2.9 Statistical analysis

The results were expressed as mean and standard deviations derived from three replicates per sample. One-way analysis of variance (followed by Tukey's test) or *t-*test was performed using SPSS 26.0 (IBM Corporation, NY, USA). A *P* value less than 0.05 indicated a significant difference. GraphPad Prism 10.0 (GraphPad Software, CA, USA) was used for plotting.

## 3 Results and discussion

### 3.1 Analysis of DF properties

**Fig. 1:**
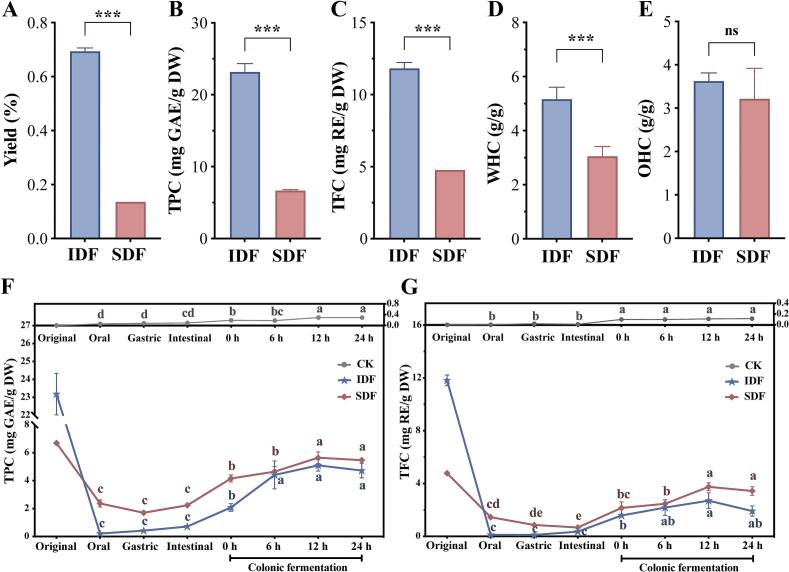
The original characteristics of SDF and IDF and the dynamic changes of bound phenolic substances released at different stages. A, Yield; B, Total phenol content (TPC); C, Total flavone content (TFC); D, Water holding capacity (WHC); E, Oil holding capacity (OHC) (Asterisks mark the significant correlations between the selected two groups: *P < 0.05; ** *P* < 0.01; *** *P* < 0.001; **** *P* < 0.0001); F, The dynamic change of total phenol content (TPC); G, The dynamic change of total flavone content (TFC). Different lowercase letters in each treatment indicate significant differences among stages (*P* < 0.05).

Grape pomace is recognized as a valuable source of DF. Fig. 1A-E illustrates the fundamental characteristics of SDF and IDF extracted from Pinot noir grape pomace. The extraction yield of IDF was 69.44 %. In contrast, SDF accounted for a significantly lower proportion of 13.61 % (Fig. 1A). As shown in Fig. 1B and C, the TPC and total flavonoid content (TFC) released from IDF were significantly higher than those from SDF, which was consistent with the findings of Xu et al. (2020). It appeared that more polyphenols and flavonoids were connected with IDF by covalent bonds. The water holding capacity (WHC) and oil holding capacity (OHC) are crucial indicators for measuring the ability of DF to retain water under external force and absorb fat, respectively (Liu et al., 2024). Our results revealed that IDF exhibited a significantly higher WHC than SDF, while both exhibited comparable OHC values (Fig. 1D, and E). This difference was mainly attributed to the effective removal of proteins, reducing sugars and starch encapsulated by IDF during enzymatic extraction, resulting in a loose, porous and irregular morphology of the rough sponge-like structure. This structural arrangement exposed more hydroxyl groups inside IDF (Ullah et al., 2017), and the hydrophilic groups of cellulose and hemicelluloses (Elleuch et al., 2011). These findings were consistent with those of Ma et al. (2021). They reported that IDF extracted from purple turnips demonstrated significantly greater water-swelling capacity than SDF.

### 3.2 Release pattern of DFP *in vitro* digestion and fermentation

#### 3.2.1 Release of TPC and TFC

The changes in TPC and TFC were measured before digestion, during digestion, and after 24 h of fermentation to assess the release behavior of bound polyphenols from IDF and SDF during simulated *in vitro* digestion and colonic fermentation. As shown in Fig. 1F and G, the initial TPC and TFC were 23.18 ± 1.16 and 11.83 ± 0.40 mg/g, respectively, in undigested IDF and 6.70 ± 0.12 and 4.78 ± 0.04 mg/g, respectively, in undigested SDF. The results demonstrated that the release ratesof TPC and TFC in both IDF and SDF were significantly higher during fermentation than during digestion, which was consistent with previous fingdings (Hou et al., 2024). This was likely because digestive enzymes had a limited ability to hydrolyze bound polyphenols, whereas gut microbiota enzymes such as esterase and xylanase facilitated their release and metabolism during colonic fermentation (Acosta-Estrada et al., 2014). The release of TPC and TFC from both IDF and SDF increased gradually throughout fermentation (0−12*h*), reaching peak levels in 12 h, followed by a slight decline thereafter. This phenomenon could be attributed to the degradation or transformation of bound polyphenols by gut microbiota, as well as their absorption and use by the body (Zhang et al., 2023). Despite the significantly higher initial TPC and TFC contents in IDF than in SDF, the cumulative release of polyphenols during fermentation was consistently higher in SDF. This might be due to the tightly packed cellulose and hemicellulose structure of IDF, which was not easily destroyed by gastrointestinal digestive enzymes and thus inhibited the release of bound polyphenols during digestion (Li et al., 2023). In contrast, the highly branched and more soluble nature of SDF facilitated the gradual release of polyphenols (Yan et al., 2024). Consequently, the release of TPC and TFC during digestion accounted for only 0.90 %–3.10 % of the total IDF content, whereas SDF exhibited a more efficient release of bioactive compounds.

#### 3.2.2 Release of monomeric phenols

**Fig. 2:**
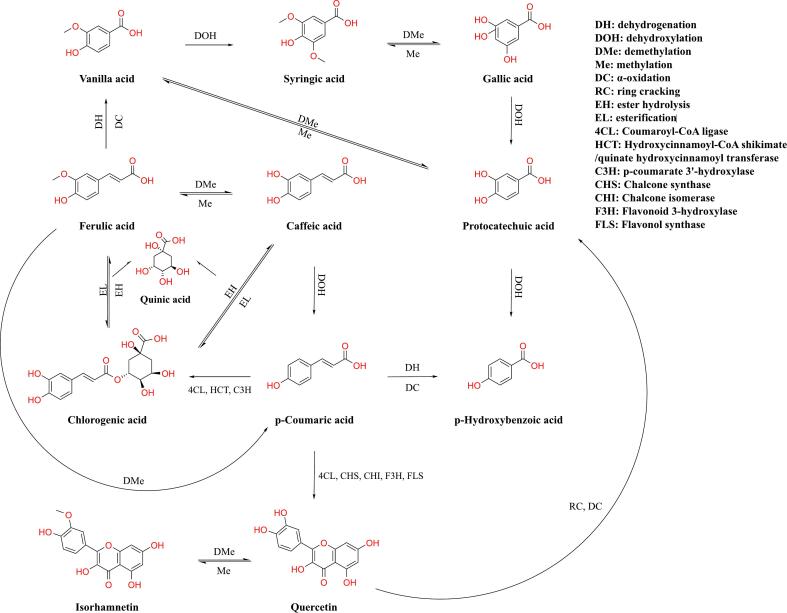
Potential metabolic pathways of monophenols released from dietary fiber combined with polyphenols.

The release, absorption, and physiological effects of polyphenols are strongly influenced by gastrointestinal digestion and gut microbiota. The structural modifications of these compounds can lead to variations in bioaccessibility and biological activity (Gao et al., 2022). Table 1 presents the release profiles of 14 monomeric phenolic compounds from IDF and SDF during *in vitro* digestion and colonic fermentation. These include five flavonoids (quercetin, catechin, epicatechin, myricetin, and isorhamnetin), one non-flavonoid (resveratrol), and eight phenolic acids (gallic acid, protocatechuic acid, gentisic acid, chlorogenic acid, vanillic acid, caffeic acid, syringic acid, and p-‌coumaric acid). Overall, the phenolic substances bound to DF were slowly and continuously released during colonic fermentation. This release amount was considerably higher than that during gastrointestinal digestion. Gentisic acid was the dominant phenolic compound released during fermentation, accounting for approximately 43.94 % and 39.16 % of the total phenolic compounds released from IDF and SDF, respectively, during the peak fermentation period. Both gentisic acid and vanillic acid exhibited a continuous release trend throughout the entire digestion and fermentation processes of IDF and SDF. However, the release of gallic acid, protocatechuic acid, and isorhamnetin peaked after 12 h of fermentation before gradually declining. This trend might be attributed to the microbial conversion of these compounds into other metabolites or non-phenolic products (Rocchetti et al., 2022). As shown in Fig. 2, ferulic acid is converted into vanillic acid through dehydrogenation and α-oxidation (Nguyen et al., 2021). Vanillic acid is then transformed into protocatechuic acid through demethylation by vanillic acid *O*-demethylase (Sánchez-Patán et al., 2012). Similarly, syringic acid can be converted into gallic acid through demethylation, which is further transformed into protocatechuic acid *via* dehydroxylation (Zhang et al., 2023). The release of isorhamnetin was also attributed to gut microbiota–mediated biotransformation of quercetin (Tamura et al., 2023).

**Table 1:** Profiles of released bound polyphenols from IDF and SDF during *in vitro* simulated digestion and colonic fermentation (μg/g).

No.	Compound	DF	Simulated digestion			Colonic fermentation			
			Oral	Gastric	Intestinal	0 h	6 h	12 h	24 h
1	Gallic acid	IDF	2.06 ± 0.04^e^	14.76 ± 2.17^d^	ND	22.19 ± 0.20^c^	26.12 ± 1.05^b^	28.86 ± 0.33^a^	21.44 ± 1.02^c^
		SDF	2.17 ± 1.02^g^	4.43 ± 0.43^f^	8.23 ± 0.10^e^	20.85 ± 0.19^c^	23.99 ± 0.05^b^	29.27 ± 0.43^a^	19.63 ± 0.21^d^
2	Protocatechuic acid	IDF	6.31 ± 0.64^c^	8.58 ± 1.25^c^	17.34 ± 0.52^b^	11.93 ± 1.40^bc^	10.23 ± 2.00^c^	28.09 ± 2.85^a^	24.03 ± 3.72^a^
		SDF	1.23 ± 0.45^e^	1.46 ± 0.34^e^	9.76 ± 1.10^d^	6.40 ± 0.70^de^	18.38 ± 0.93^c^	35.39 ± 2.21^a^	28.75 ± 4.62^b^
3	Gentian acid	IDF	1.29 ± 0.26^d^	10.17 ± 0.91^d^	7.42 ± 0.83^d^	73.02 ± 3.21^c^	185.58 ± 8.21^b^	186.58 ± 6.72^b^	203.65 ± 8.74^a^
		SDF	ND	5.48 ± 0.28^c^	4.57 ± 0.58^c^	176.59 ± 3.21^b^	190.65 ± 6.12^ab^	199.35 ± 6.12^a^	200.80 ± 10.00^a^
4	Chlorogenic acid	IDF	ND	ND	ND	13.00 ± 3.12^b^	12.94 ± 0.15^b^	20.91 ± 0.98^a^	11.98 ± 0.83^b^
		SDF	ND	ND	ND	15.36 ± 3.01^a^	13.20 ± 0.36^a^	12.84 ± 0.32^a^	12.33 ± 3.14^a^
5	Vanillic acid	IDF	3.88 ± 0.28^c^	11.77 ± 0.17^b^	11.29 ± 0.45^b^	21.20 ± 2.57^a^	7.84 ± 1.56^bc^	21.59 ± 1.65^a^	23.39 ± 5.09^a^
		SDF	1.89 ± 0.16^c^	ND	2.69 ± 0.10^c^	8.31 ± 0.40^b^	8.99 ± 2.89^b^	5.91 ± 0.41^b^	19.60 ± 2.59^a^
6	Caffeic acid	IDF	0.81 ± 0.03^c^	1.86 ± 0.14^b^	5.72 ± 0.21^a^	ND	ND	ND	ND
		SDF	ND	ND	ND	ND	ND	ND	ND
7	Syringic acid	IDF	5.40 ± 0.96^c^	23.54 ± 0.57^b^	26.09 ± 1.48^b^	6.54 ± 2.89^c^	25.92 ± 0.60^b^	25.93 ± 3.91^b^	34.79 ± 2.75^a^
		SDF	0.35 ± 0.10^a^	ND	0.50 ± 0.24^a^	ND	ND	ND	ND
8	Catechin	IDF	ND	ND	ND	ND	24.05 ± 0.07^c^	56.99 ± 6.22^a^	48.85 ± 1.46^b^
		SDF	ND	ND	ND	ND	ND	30.19 ± 2.19^b^	86.29 ± 9.44^a^
9	Epicatechin	IDF	ND	ND	ND	ND	ND	ND	ND
		SDF	ND	ND	ND	ND	ND	ND	85.50 ± 7.73
10	p-‌Coumaric acid	IDF	1.06 ± 0.01^c^	2.09 ± 0.02^c^	ND	13.73 ± 1.02^a^	12.51 ± 0.11^ab^	11.78 ± 0.77^b^	11.02 ± 1.02^b^
		SDF	2.32 ± 0.06^d^	5.43 ± 0.83^c^	7.24 ± 0.39^c^	12.95 ± 1.04^a^	10.39 ± 0.22^b^	12.72 ± 1.02^a^	11.19 ± 0.64^ab^
11	Myricetin	IDF	ND	ND	ND	ND	ND	17.26 ± 4.14	ND
		SDF	ND	ND	ND	36.32 ± 0.85^a^	28.31 ± 3.28^b^	ND	ND
12	Quercetin	IDF	ND	7.78 ± 0.78^d^	13.67 ± 0.43^d^	ND	33.10 ± 2.51^c^	41.10 ± 0.89^b^	49.66 ± 5.65^a^
		SDF	12.62 ± 0.99^a^	14.62 ± 2.73^a^	17.53 ± 1.29^a^	ND	ND	ND	ND
13	Isorhamnetin	IDF	1.56 ± 0.20^d^	4.12 ± 0.84^c^	6.36 ± 1.68^c^	6.29 ± 0.79^c^	25.69 ± 0.42^a^	22.77 ± 1.69^ab^	18.81 ± 5.51^b^
		SDF	1.35 ± 0.25^e^	1.66 ± 1.03^e^	10.16 ± 0.75^d^	24.32 ± 1.54^b^	22.21 ± 2.25^b^	34.90 ± 1.60^a^	17.34 ± 2.16^c^
14	Resveratrol	IDF	ND	ND	ND	1.16 ± 0.04	ND	ND	ND
		SDF	ND	ND	ND	ND	ND	2.68 ± 0.90	ND
	Sums	IDF	22.93 ± 2.05^f^	85.65 ± 2.86^e^	89.93 ± 2.62^e^	190.90 ± 4.32^d^	389.97 ± 6.48^c^	480.24 ± 13.68^a^	463.45 ± 1.90^b^
		SDF	25.76 ± 1.69^g^	39.75 ± 2.63^f^	70.71 ± 4.01^e^	328.25 ± 5.71^d^	351.14 ± 15.94^c^	398.76 ± 0.89^b^	512.77 ± 10.69^a^

Different letters on each line represent significant differences (*P* < 0.05). ND: not detected.

Moreover, significant differences were observed in the release patterns of certain monomeric phenols between IDF and SDF. For instance, syringic acid and quercetin showed a significantly increasing release trend in IDF, whereas they were detected only during the digestion phase of SDF. This suggested that the rigid structure of IDF protected bound polyphenols from degradation, allowing their gradual release during fermentation. Conversely, the loose and soluble nature of SDF facilitated the rapid release and absorption of certain phenolic compounds. Vanillic acid is converted into syringic acid through a dihydroxylation process (Zhang et al., 2023). Quercetin can be produced using *p*-coumaric acid as a precursor (Marin et al., 2018). It is metabolized rapidly. Additionally, the biotransformation and absorption of its glycoside moieties mainly occur in the small intestinal epithelial cells; only a small portion entering the colon is transformed into other molecules such as 3,4-dihydroxyphenylacetic acid and protocatechuic acid (Tang et al., 2016). Similarly, caffeic acid, which is obtained through ferulic acid demethylation (Zhang et al., 2019), is almost completely absorbed by the small intestine (Tomas-Barberan et al., 2014), explaining why caffeic acid was not detected during fermentation.

However, chlorogenic acid was detected only during the fermentation phase, and its content remained relatively stable throughout the fermentation process. This might be because the precursor of chlorogenic acid, p-coumaric acid (He et al., 2013), is not released during the digestion phase and is instead released in large amounts at the beginning of fermentation (0 h), where it is then converted into chlorogenic acid. Additionally, chlorogenic acid is rarely hydrolyzed in the stomach, can escape absorption in the small intestine, and exhibits no significant bioavailability before.

reaching the colon (Song et al., 2019). Myricetin, resveratrol, and epicatechin were only occasionally detected in small amounts during the fermentation phase, indicating that these compounds can be converted into other compounds by the colonic microbiota (Farias et al., 2025). Catechin was only significantly captured in the later stage of fermentation. This might be due to its strong affinity to DF, which allowed it to bypass digestion and directly enter the colon almost intact (Li & Van de Wiele, 2023). The type and abundance of monophenols and their decomposition products also rely on the diversity of gut microbiota (Li et al., 2022). Therefore, the mechanism underlying changes in phenolic compound catabolism needs further exploration.

### 3.3 Potential bioactivity of DFP *in vitro* digestion and fermentation

#### 3.3.1 DPPH, ABTS, and hydroxyl radical–scavenging abilities

**Fig. 3:**
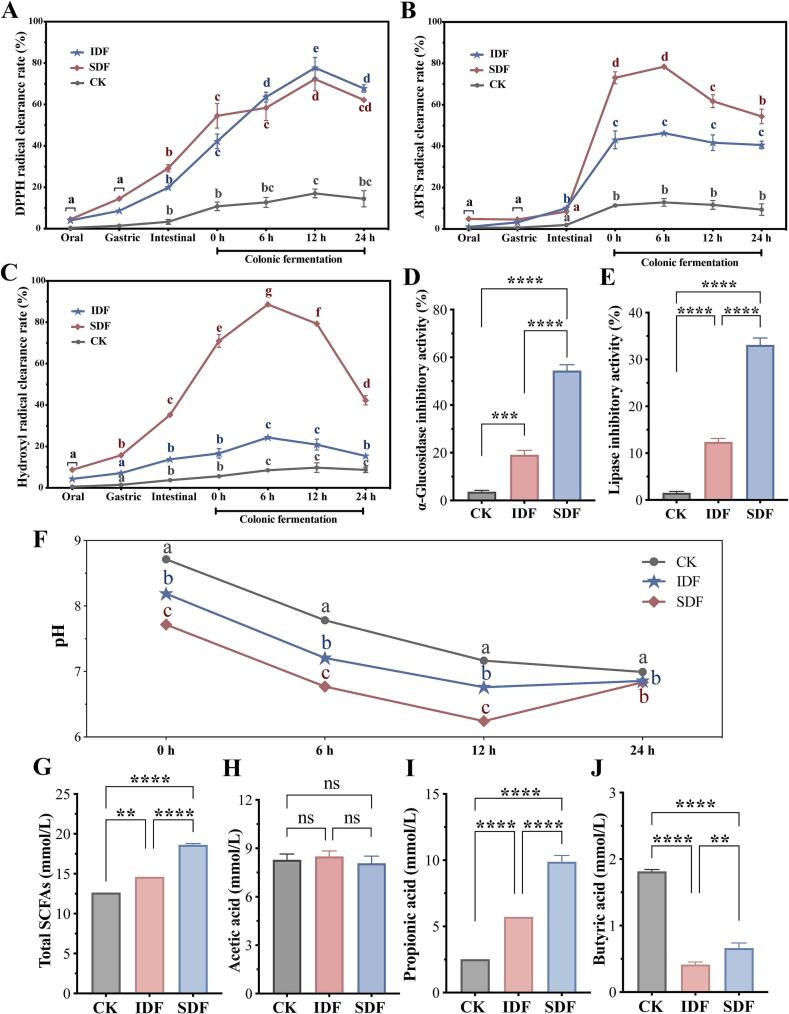
The changes in antioxidant activities of IDF and SDF at each stage of digestion and fermentation (A, DPPH radical scavenging activity; B, ABTS radical scavenging activity; C, hydroxyl radical scavenging activity) (Different lowercase letters in each treatment indicate significant differences among stages (*P* < 0.05)), inhibition rate of enzyme activity in the intestinal digestion stage (D, α -glucosidase inhibition activity; E, lipase inhibitory activity), and the changes of pH value during colonic fermentation (F) (Different lowercase letters under each fermentation stage indicate significant differences among treatments (*P* < 0.05)) and the levels of total SCFAs (G), acetic acid (H), propionic acid (I) butyric acid (J) after colonic fermentation for 24 h. Asterisks mark the significant correlations between the selected two groups: *P < 0.05; ** *P* < 0.01; *** *P* < 0.001; **** *P* < 0.0001.

Polyphenols undergo biotransformation and use during digestion and intestinal fermentation. They mainly exhibit strong free radical–scavenging ability by donating electrons or hydrogen atoms, thereby reducing oxidative damage (Pastoriza et al., 2011). We focused on DPPH, ABTS, and hydroxyl radical–scavenging ability to evaluate the antioxidant activity of DFP. The antioxidant activity of DFP was significantly higher during colonic fermentation than during gastrointestinal digestion (Fig. 3A–3C). This aligned with the notion that DFs resist gastrointestinal digestion and absorption, thereby limiting polyphenol release and bioavailability (Lattimer & Haub, 2010). As DF fermentation progresses, polyphenols are gradually released or transformed into metabolites with higher antioxidant activity through microbial processes including ring cleavage, ester bond hydrolysis, demethylation, dehydroxylation, dehydrogenation, and deglycosylation (Rocchetti et al., 2022). For example, microbes can transform quercetin into protocatechuic acid and 3,4-dihydroxyphenylacetic acid, which exhibit strong DPPH radical–scavenging and superoxide dismutase–like activities (Tang et al., 2016).

The DPPH radical–scavenging activity peaked after 12 h of fermentation before declining, whereas ABTS and hydroxyl free radical–scavenging activities started declining after 6 h of fermentation. This decrease was likely due to the gradual degradation or metabolic transformation of phenolic substances into less potent antioxidants, consistent with previous findings (Hou et al., 2024). DPPH is the most stable and is reduced by hydrogen supply from antioxidants; ABTS^·+^ is reduced by hydrogen supply and electron transfer; and hydroxyl free radicals are more active and are reduced by the phenolic hydroxyl (–OH) group of polyphenols *via* hydrogen supply or electron transfer (Ambigaipalan et al., 2015). The observed differences in antioxidant capacity may also result from the structural changes in polyphenols during fermentation, thus altering their antioxidant mechanisms. Moreover, SDF exhibited high ABTS and hydroxyl free radical–scavenging activities throughout colonic fermentation, which might be related to the presence of more SCFAs and phenolic compounds.

#### 3.3.2 Inhibitory effects on α-glucosidase and lipase

α-Glucosidase is a key enzyme in carbohydrate metabolism that hydrolyzes carbohydrates into glucose, thereby elevating postprandial blood glucose levels (Huamán-Castilla et al., 2021). Lipase is a key enzyme involved in lipid metabolism, and polyphenols have been shown to inhibit lipase activity, thereby limiting fat digestion (Buchholz & Melzig, 2015). Considering that α-glucosidase and lipase primarily function in the intestine, we evaluated the inhibitory effect of bound polyphenols released during intestinal digestion on these enzymes (Fig. 3D and E) as an indicator of hypoglycemia and hypolipemia, respectively. Both IDF and SDF significantly inhibited α-glucosidase and lipase activities in the intestinal digestion phase (*P* < 0.05). The enzyme-inhibitory effects of polyphenols are primarily due to the noncovalent interactions. Specifically, the phenolic hydroxyl group forms hydrogen bonds with the active sites of the enzyme, and the benzene ring of the polyphenols can form π-π conjugation with tryptophan and tyrosine in the enzyme (Sun et al., 2020). Moreover, the noncovalent interaction between free polyphenols with gallic acid groups and enzymes is stronger (Cao et al., 2020). This indicates that the inhibition rate of α-amylase activity highly depends on the polyphenol structure provided by DFs. You et al. (2012) proposed that the phenolic inhibition patterns of α-glucosidase and lipase in muscadine berries and seeds are both competitive, with quercetin considered as a major contributor to α-glucosidase inhibition. Yu et al. (2025) considered that the combination of polyphenols also had a dose-dependent inhibitory effect on α-amylase and α-glucosidase activities. Our results suggested that SDF demonstrated significantly stronger inhibitory effects than IDF (*P* < 0.05). This was possibly due to its higher polyphenol content (including gallic acid and quercetin) in the intestinal digestion stage and superior solubility and dispersibility, which facilitated better interaction with digestive enzymes.

### 3.4 pH regulation and SCFA production during the colonic fermentation of DF

pH regulation in the gut is crucial for maintaining the balance of the community composition of gut microbiota and promoting the production of beneficial microbial metabolites (Yin et al., 2020). As shown in Fig. 3F, the pH in all treatment groups gradually decreased within 12 h. At each time point, SDF exhibited the lowest pH, followed by IDF and then the control (CK) (*P* < 0.05). This suggested that both IDF and SDF helped regulate intestinal pH, with SDF playing a more significant role. The pH in the CK group continued to decline after 12 h of fermentation, the pH of IDF stabilized, and the pH of SDF began to rise slightly. All groups exhibited a pH value of 6.90 ± 0.08 after 24 h of fermentation. The continuous decline in the pH in the CK group was likely a result of protein fermentation, whereas the reduced pH in the IDF and SDF groups compared with the CK group was related to phenolic metabolism and SCFA production (Saura-Calixto et al., 2010).

SCFAs are functional biomarkers produced through the microbial fermentation of DFP. They, including acetic acid, propionic acid, and butyric acid, are key metabolites serving as the primary source of energy for colonic cells. They play a role in gut health by supporting immune function, inhibiting the growth of pathogenic microorganisms, and reducing inflammation (colitis and carcinogenesis) (Liu et al., 2021). Our results showed that DF significantly increased the total SCFA and propionic acid contents, with SDF producing the highest levels (*P*  <  0.05; Fig. 3G–3J). Among these, propionic acid was the key contributor to the observed significant differences, accounting for 53.10 % of total SCFAs in the SDF group. Although butyric acid contents were the highest in the CK group, excessive butyrate concentrations (>1 mmol/L) might impair enteric nerve cells, leading to functional constipation (Wang et al., 2023). These findings suggested that the interaction between DF and polyphenols promoted SCFA production during *in vitro* fermentation while maintaining an optimal concentration for gut health, with SDF demonstrating the most pronounced effect.

### 3.5 Modulation of gut microbiota by DF

#### 3.5.1 Diversity of intestinal microbiota

**Fig. 4:**
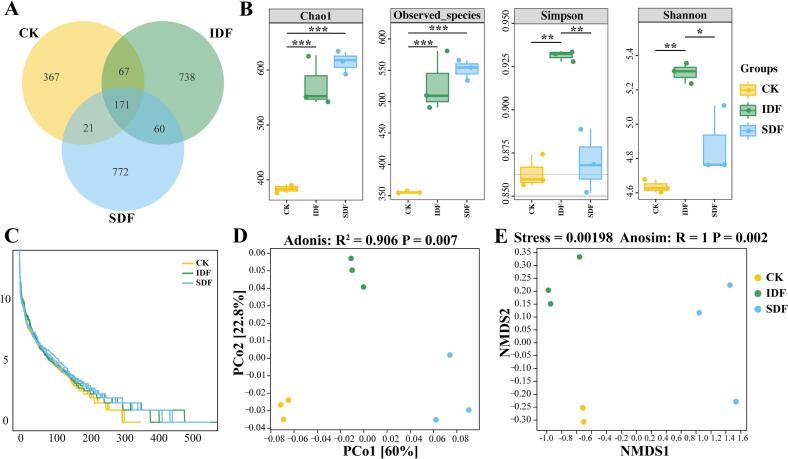
Effect of IDF and SDF on the diversity of gut microbiota. A, The venn diagram; B, The α-diversity plot based on Chao1, Observed_species, Simpson and Shannon index; C, Rank abundance curve; D, The β-diversity plot based on PCoA and Adonis analysis; E, The β-diversity plot based on NMDS and Anosim analysis.

16S rRNA high-throughput sequencing technology was used to analyze the composition of intestinal flora regulated by IDF and SDF after enter colon fermentation. Fig. 4A presents the differences in ASVs across various treatment groups. The unique ASV counts for each group (CK: 367; IDF: 738; SDF: 772) were significantly higher than the shared ASVs among the three groups (171), indicating that DF supplementation significantly influenced species richness and composition. The rank abundance curve in the community analysis can directly reflect the high abundance and counts of rare ASVs in the community. As shown in Fig. 4C, the IDF and SDF groups exhibited higher species richness and a more balanced community structure than the CK group after colonic fermentation. Subsequently, Chao1, Observed_species, Simpson, and Shannon indices were calculated to more accurately quantify species richness and community diversity (Fig. 4B). The results revealed that the species richness in the CK group was significantly lower than that in the DF group; IDF supplementation significantly enhanced overall community diversity (*P* < 0.05). Following this, we used the weighted_unifrac distance of the PCoA model with Adonis analysis and the Bray–Curtis's distance of the NMDS model with Anosim analysis to evaluate the differences in β-diversity of the gut microbiota between the IDF, SDF, and CK groups (Fig. 4D and E). The clear separation among the three groups indicated that IDF and SDF significantly altered the structure and composition of gut microbiota differently (PCoA: *R*^2^ = 0.906, *P* = 0.007; NMDS: Stress = 0.00198, *R* = 1, *P* = 0.002). In conclusion, IDF and SDF significantly influenced β-diversity among gut microbiota and notably enhanced the species richness of gut microbiota. IDF contributed the most to enriching the α-diversity of the microbial community.

#### 3.5.2 Composition of intestinal microbiota species

**Fig. 5:**
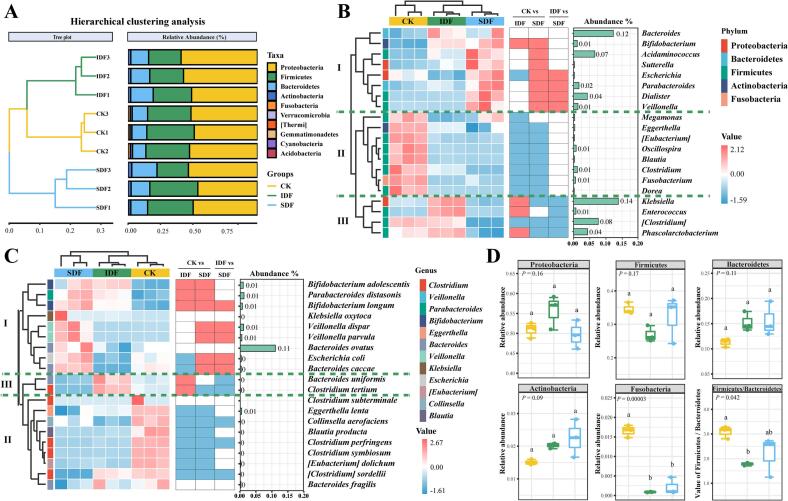
IDF and SDF effects on the species composition of gut microbiota. A, dendrogram clustering analysis and relative abundance of the top 10 bacterial phyla; B, heatmap of bacterial classification profiles based on the top 20 dominant genera (The colour blocks on the right side of the heatmap represent the significance results of pairwise comparisons between groups, with red representing a significant increase and blue representing a significant decrease); C, heatmap of bacterial classification profiles based on the top 20 dominant species; D, represents the relative abundance of Proteobacteria, Firmicutes, Bacteroidetes, Actinobacteria, Fusobacteria under different treatments, and the value of Firmicutes/Bacteroidetes. (For interpretation of the references to colour in this figure legend, the reader is referred to the web version of this article.)

The analyses were conducted at the phylum, genus, and species levels to further clarify the impact and difference of IDF and SDF on the composition of intestinal flora. The UPGMA clustering analysis based on the Bray–Curtis distance matrix showed that the clusters were significantly separated among different treatment groups, confirming that DF supplementation remarkably altered the species and relative abundance of dominant bacteria among the intestinal flora (Fig. 5A). At the phylum level, the dominant bacterial phyla across all groups were Proteobacteria, Firmicutes, Bacteroidetes, Actinobacteria, and Fusobacteria. Both IDF and SDF significantly reduced the relative abundance of Fusobacteria (*P* < 0.0001). They decreased the Firmicutes/Bacteroidetes ratio, with IDF exhibiting a more pronounced effect (*P* < 0.05) (Fig. 5D). Fusobacteria is closely related to the occurrence of colorectal tumors (Nakatsu et al., 2025). Obesity, diabetes, and inflammatory bowel disease are all associated with elevated Firmicutes/Bacteroidetes ratios in the gut microbiota (Yañez et al., 2021). Fig. 5B and C shows the effects of DF on the dominant gut flora at the top 20 bacterial genus level and species level, respectively, in the form of a clustering heatmap. As shown in the figure, the heatmap is divided into three clustering blocks (I, II, and III). Clusters I, II, and III represent the high-abundance bacteria enriched in the SDF, CK, and IDF groups, respectively. The linear discriminant analysis effect size identified differentially enriched bacteria among the groups (Fig. S2). At the genus level, 24 differentially marked genera were identified across all groups. Among them, 11 genera ranked among the top 20 in relative abundance, and were group-specific: CK group included *Clostridium*, *[Clostridium]*, *Oscillospira*, and *Megamonas*; IDF group included *Klebsiella* and *Phascolarctobacterium*; and SDF group included *Acidaminococcus*, *Dialister*, *Veillonella*, *Sutterella*, and *Escherichia*. These genera contributed most significantly to the differentiation in microbial community composition among the groups. We also found that the relative abundance of the dominant bacteria was higher in the DF group than in the CK group. This indicated that the presence or absence of DF significantly changed the composition of the dominant gut microbiota and occupied the main dominant position.

Compared with CK, both IDF and SDF significantly elevated the relative abundance of *Bifidobacterium* (*P* < 0.05) (Fig. 5B). *Bifidobacterium* plays a crucial role in gut health by inhibiting pathogenic colonization in the colon, reducing inflammation, promoting antioxidant activities, maintaining intestinal homeostasis, and improving immunity (Li et al., 2025). Compared with CK, SDF significantly increased the relative abundance of *Acidaminococcus*, *Sutterella*, *Parabacteroides*, *Dialister*, and *Veillonella* (*P* < 0.05). Of these, *Parabacteroides*, *Acidaminococcus*, *Dialister,* and *Veillonella* are known to produce SCFAs (Fontdevila et al., 2024; Zhang et al., 2025). Moreover, *Clostridium*, *Eubacterium*, and *Fusobacterium* are genera crucial for fermenting undigested DFs to produce butyrate in the colon (Jiang et al., 2018). Excessive butyrate production in the CK group might be a result of the significant enrichment of these genera in this group. Furthermore, the abundance of harmful genera such as *Eggerthella* (linked to inflammatory bowel disease and autoimmune disorders) and *Blautia* (associated with obesity, diabetes, and nonalcoholic steatohepatitis) was significantly reduced in both IDF and SDF groups (*P* < 0.05) (Fig. 5B**)** (Alexander et al., 2022; Zhao et al., 2021). At the species level, both IDF and SDF significantly increased the abundance of *Bifidobacterium longum*, *Bifidobacterium adolescentis*, and *Parabacteroides distasonis* compared with CK (*P* < 0.05) (Fig. 5C). SDF significantly promoted the growth of *Escherichia coli* and *Bacteroides caccae*, whereas IDF inhibited it. Of these, *B. longum* and *B. adolescentis* regulated colonic inflammation and inhibited cancer cell growth (Kim et al., 2025). *P. distasonis* activated intestinal gluconeogenesis, regulated appetite, promoted liver glycogen synthesisin the liver, and prevented glucose metabolism disorders (Wang et al., 2019).

These findings indicated that DFs, particularly SDF, significantly modulated the diversity and composition of gut microbiota by promoting the growth of beneficial bacteria in the gut while suppressing the growth of harmful bacteria. This complex relationship between gut bacteria and bound polyphenols needs further exploration.

#### 3.5.3 Functional prediction of gut microbiota

**Fig. 6:**
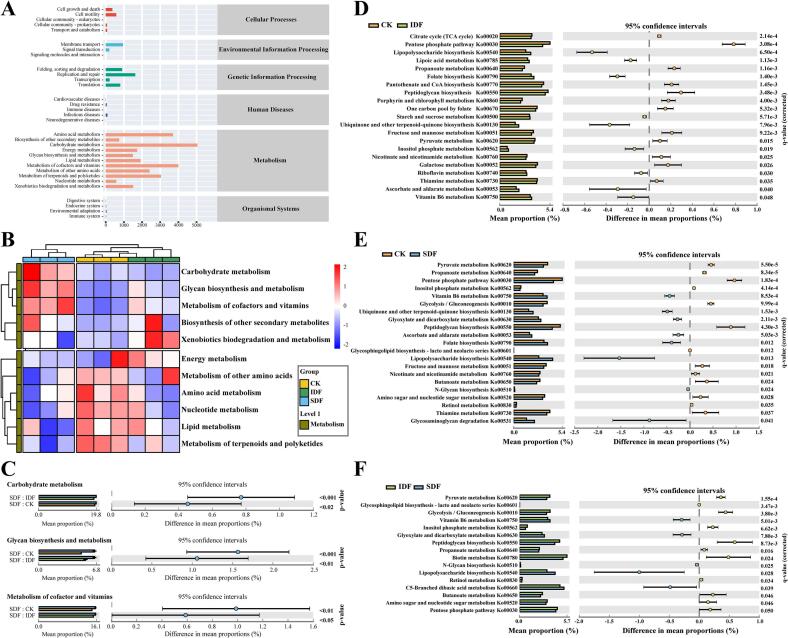
Effects of IDF and SDF on the metabolic functions of gut microbiota. A, Abundance of level 2 functional pathways under six level 1 metabolic pathways in the KEGG database; B, PICRUSt-predicted metabolic pathway clustering heatmap of gut microbiota at Level 2; C, Intergroup differences in Carbohydrate metabolism, Glycan biosynthesis and metabolism, and Metabolism of cofactor and vitamins; D, Metabolic functions with significant differences in CK *vs.* IDF; E, Metabolic functions with significant differences in CK *vs.* SDF; F, Metabolic functions with significant differences in IDF *vs.* SDF.

PICRUSt2 was employed to predict the functions of gut bacteria through the Kyoto Encyclopedia of Genes and Genomes (KEGG) metabolic pathways. The metabolism-related sequence abundance was as high as 81.3 % (Fig. 6A). Further clustering at metabolic Level 2 revealed significant differences in metabolic functions between the SDF group and the remaining two groups (Fig. 6B). The STAMP analysis identified metabolic pathways that differed significantly between the groups (Fig. 6C). At KEGG Level 2, carbohydrate metabolism, glycan biosynthesis and metabolism, and metabolism of cofactor and vitamins pathways were significantly enriched in the SDF group compared with the other two groups (*P* < 0.05). Accordingly, the differences in metabolic functions (KO and KEGG orthologous groups) corresponding to Level 3 under the aforementioned three Level 2 metabolic pathways were analyzed (Fig. 6D–6F). Compared with CK, the combination of IDF and SDF significantly enhanced vitamin B6, ascorbate, and aldarate metabolism; promoted the biosynthesis of lipopolysaccharide, folate, ubiquinone, and other terpenoid quinones; and inhibited propanoate metabolism and the pentose phosphate pathway (*P* < 0.05) (Fig. 6D and E). Moreover, the differences in DF types also selectively affected the metabolic pathways of gut microbiota (Fig. 6F).

The inhibition of propionate metabolism and pentose phosphate pathway in carbohydrate metabolism by DF explains why the content of propionic acid was significantly higher in the DF group than in the CK group, which also helped to slow down the rate of glycolysis and maintain stable blood sugar levels. Similar to our study, another study found that *Bifidobacterium* produced carbohydrate lyase to break down DF and regulate glucose and lipid metabolism during fermentation (Jiao et al., 2025). Glycan biosynthesis and metabolism are the main functions of the intestinal microbiota, which are responsible for metabolizing hard-to-degrade polysaccharides into SCFAs (Koropatkin et al., 2012). The catabolism of B vitamins can provide energy for metabolism in the body, whereas anabolism produces key cofactors influencing bacterial metabolism and immune regulation processes (Hanna et al., 2022). The ascorbate and aldarate metabolism upregulated by DF is an essential pathway of carbohydrate metabolism, which protects cells from oxidative damage and enhances antioxidant activity (Peng et al., 2023). Therefore, DF and polyphenols influence metabolic pathways by changing the intestinal flora; however, the exact metabolic mechanism remains to be elucidated.

### 3.6 Regulatory effect of DF on the intestinal microenvironment

**Fig. 7:**
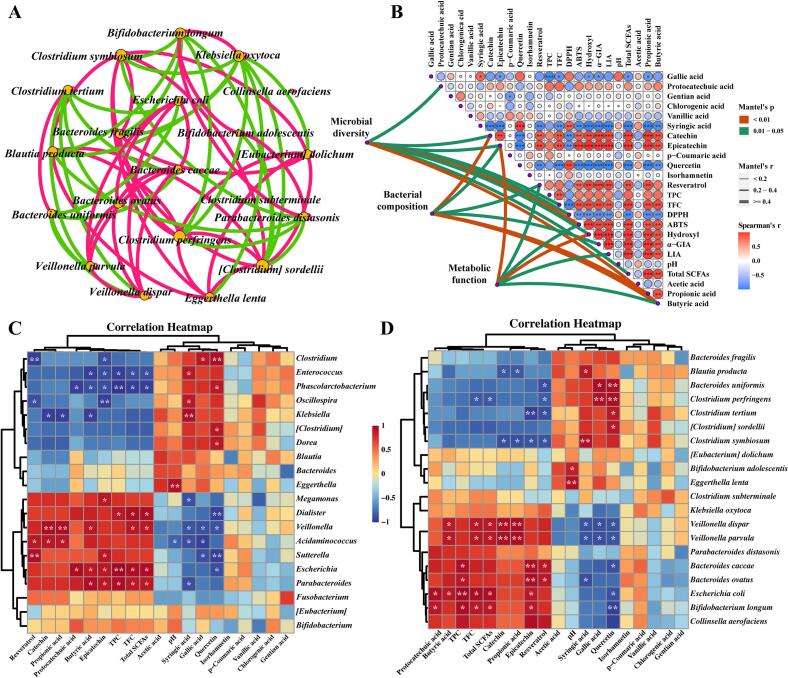
Correlation analysis of intestinal microbiota and environmental parameters. A, Co-occurrence network of Top20 species (filter condition: |r| ≥ 0.6 and *P*<0.05; node size represents degree; red line and green line indicate positive and negative connections between nodes, respectively); B, Mantel test (based on Spearman) of intestinal microbiota diversity, species composition, metabolic function with environmental parameters; C, Correlation clustering heatmap of Top20 genera with intestinal environmental parameters; D, Correlation clustering heatmap of Top20 species with intestinal environmental parameters. Abbreviations: TPC, Total phenol content; TFC, Total flavone content; α-GIA, α-Glucosidase inhibitory activity; LIA, Lipase inhibitory activity. (For interpretation of the references to colour in this figure legend, the reader is referred to the web version of this article.)

The interactions between the dominant bacterial species were evaluated by establishing a co-occurrence network (Fig. 7A). *B. longum*, *B. adolescentis*, and *P. distasonis* exhibited pairwise positive correlations. The Mantel test was conducted to assess the relationship between gut microbiota diversity, species composition, metabolic functions, and gut environmental parameters (Fig. 7B). Overall, the antioxidant activity index (except for DPPH) exhibited an extremely strong correlation with microbial diversity and metabolic function. Epicatechin and butyric acid demonstrated relatively strong correlations with microbial diversity, species composition, and metabolic function. Plant compounds with poor bioavailability, such as catechin, are eventually transformed into metabolites with improved bioavailability and biological activity through microbial fermentation (Xiao et al., 2022). Significant positive correlations were observed between some monophenols (catechin, epicatechin, and resveratrol), TFC, ABTS and hydroxyl radical–scavenging capacity, α-glucosidase-inhibitory activity, lipase-inhibitory activity, total SCFAs, and propionic acid. In contrast, significant negative correlations were found between these parameters and syringic acid, quercetin, and DPPH (*P* < 0.05).

The relationship between microbial communities and the intestinal microenvironment was further elucidated by performing a Spearman's rank correlation coefficient analysis to examine the correlation between the top 20 bacterial genera and species with phenolic compounds, pH, and SCFAs (Fig. 7C and D). *Escherichia* displayed similar results with several beneficial genera including *Dialister*, *Veillonella*, *Acidaminococcus*, *Sutterella*, and *Parabacteroides* under the premise of taking up DFP. Their relative abundance was positively correlated with the contents of TFC, TPC, some monomeric phenols (catechin, epicatechin, and resveratrol), total SCFAs, propionic acid, and butyric acid in the fermentation broth (Fig. 7C). Similar to our results, Yang et al. (2024) also found that the abundance of *Acidaminococcus* was positively correlated with the contents of combined polyphenols such as catechins, suggesting that it might have a strong ability to degrade combined polyphenols. *Parabacteroides*, *Acidaminococcus*, *Dialister*, and *Veillonella* have been reported to enrich the role of SCFAs, supporting our results (Fontdevila et al., 2024; Zhang et al., 2025). At the species level (Fig. 7D), the relative abundances of *Veillonella dispar* and *V. parvula* were significantly positively correlated with the contents of butyric acid, propionic acid, and total SCFAs, further confirming their contribution to SCFA production (*P* < 0.05). In addition, some flavanol monomers such as protocatechuic acid and epicatechin could significantly enrich *B. longum* and *E. coli* (*P* < 0.05). Epicatechin, catechin, and resveratrol significantly inhibited the growth of *Clostridium symbiosum* (*P* < 0.05). The abundance of *B. caccae* and *Bacteroides ovatus* was significantly positively correlated with TPC and epicatechin content (*P* < 0.05). In summary, the combined effect of DF and polyphenols promoted the proliferation of beneficial bacteria while inhibiting the growth of harmful bacteria.

## 4 Conclusions

This study demonstrated that the SDF and IDF of Pinot noir grape pomace had strong biological activity, but their functional properties were differentiated. Overall, the release of bound polyphenols and antioxidant activity during colon fermentation was significantly higher than that in the gastric–intestinal digestion stage. SDF exhibited superior TPC/TFC release efficiency and stronger ABTS/hydroxyl radical–scavenging ability, α-glycosidase-inhibitory activity, and lipase-inhibitory activity, whereas IDF preferentially enriched microbial α-diversity. Both fractions regulated gut microbiota composition by reducing the Firmicutes/Bacteroidetes ratio by 29.4 %–43 %), promoting the growth of probiotics (*Bifidobacterium*), and suppressing the growth of pathogens (*Eggerthella*, *Clostridium*, and *Blautia*). The metabolic pathway analysis identified shared activation of vitamin B6 metabolism, ascorbate–aldarate pathways, and lipopolysaccharide–folate biosynthesis. The synergistic effects of the fiber–polyphenol complex system on SCFA production (1.3- to 2.9-fold increase for propionate) and pH modulation highlight their prebiotic potential. However, the mechanistic interactions between DF components and microbiota require further investigation.

## CRediT authorship contribution statement

**Yuanyuan Li:** Writing – original draft, Visualization. **Xiaoxue Chen:** Validation, Software. **Gongda Wang:** Data curation. **Linting Xu:** Investigation. **Yichen Liu:** Validation. **Chunlong Yuan:** Writing – review & editing, Supervision. **Junjun Li:** Supervision, Project administration.

## Informed consent statement

All volunteers signed an informed consent form before the experiment.

## Institutional review board statement

The research was carried out in line with the ethical procedure guidelines mandated by Northwest A&F University.

## Declaration of competing interest

The authors declare that they have no known competing financial interests or personal relationships that could have appeared to influence the work reported in this paper.

## Data Availability

The authors do not have permission to share data.

## Appendix A Supplementary data

Supplementary materials Image 1
