# Supplementary material for: The release patterns and potential prebiotic characteristics of soluble and insoluble dietary fiber-bound polyphenols from pinot noir grape pomace in vitro digestion and fermentation
Source: Food Chem X. 2025 Jun 21;29:102694. doi: 10.1016/j.fochx.2025.102694 (PMC12242010; doi:10.1016/j.fochx.2025.102694)
Supplement: Supplementary file 1 — Supplementary materials [file mmc1.docx]

**Supplementary Data**

**Figure S1.** Schematic diagram of the main steps and sample collection of the simulated *in vitro* digestion and colonic fermentation.

**
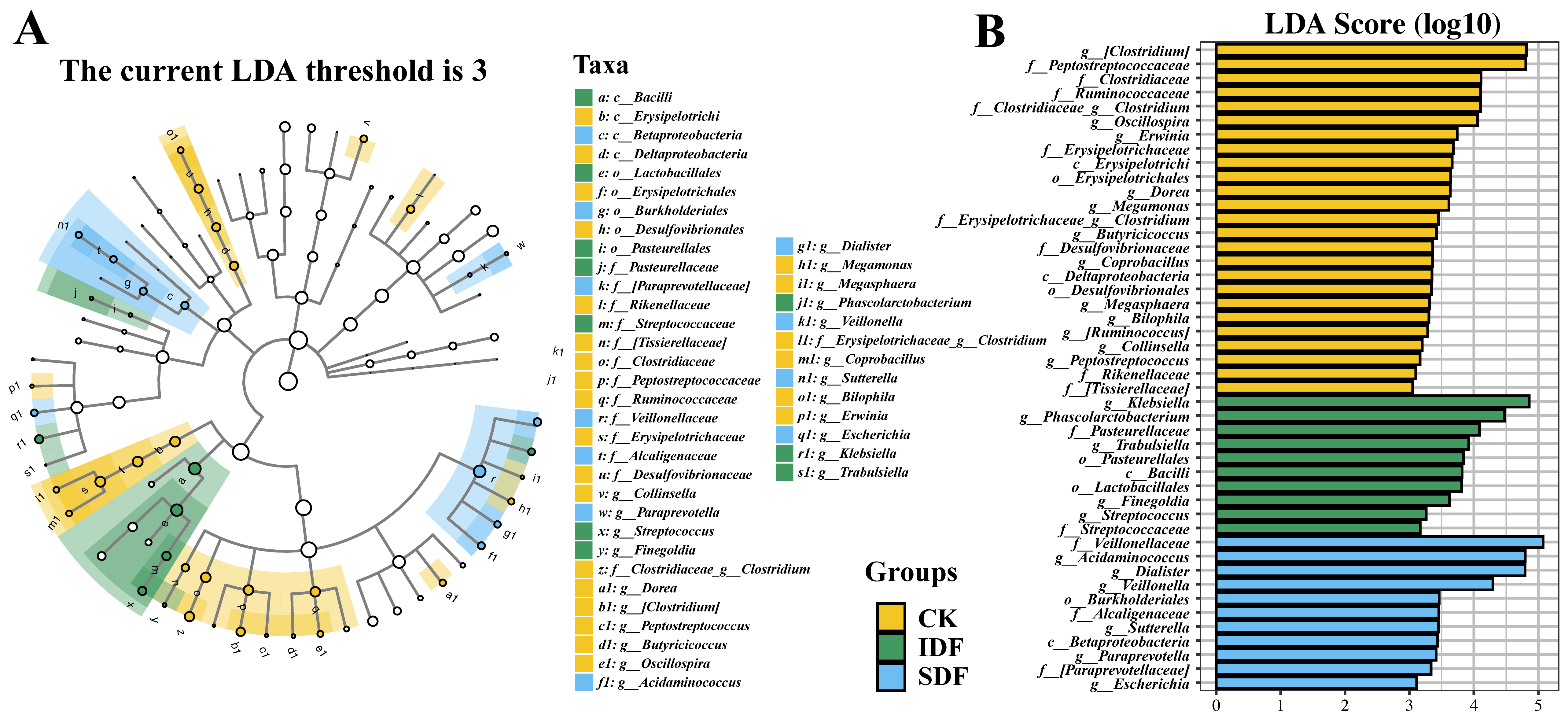
**

**Figure S2.** Differentially enriched bacteria were identified by Linear discriminant analysis (LDA) effect size (LEfSe). A, Evolutionary branching diagram of LEfSe analysis; B, Histogram of differential bacteria with LDA value (log 10) higher than 3.
